# Image analysis pipeline for segmentation of a biological porosity network, the lacuno-canalicular system in stingray tesserae

**DOI:** 10.1016/j.mex.2020.100905

**Published:** 2020-05-01

**Authors:** Merlind Schotte, Júlia Chaumel, Mason N. Dean, Daniel Baum

**Affiliations:** a Visual Data Analysis Department*,* Zuse Institute Berlin, Takustrasse 7*,* 14195 Berlin, Germany; b Department of Biomaterials*,* Max Planck Institute of Colloids and Interfaces*,* Am Mühlenberg 1*,* 14476 Potsdam, Germany

**Keywords:** Image segmentation, Watershed algorithm, Cavity segmentation, Distance-based separation, Porosity network, Lacuno-canalicular system, Synchrotron microCT data

## Abstract

A prerequisite for many analysis tasks in modern comparative biology is the segmentation of 3-dimensional (3D) images of the specimens being investigated (e.g. from microCT data). Depending on the specific imaging technique that was used to acquire the images and on the image resolution, different segmentation tools are required. While some standard tools exist that can often be applied for specific subtasks, building whole processing pipelines solely from standard tools is often difficult. Some tasks may even necessitate the implementation of manual interaction tools to achieve a quality that is sufficient for subsequent analysis. In this work, we present a pipeline of segmentation tools that can be used for the semiautomatic segmentation and quantitative analysis of voids in tissue (i.e. internal structural porosity). We use this pipeline to analyze lacuno-canalicular networks in stingray tesserae from 3D images acquired with synchrotron microCT.

• The first step of this pipeline, the segmentation of the tesserae, was performed using standard marker-based watershed segmentation.
• The efficient processing of the next two steps, that is, the segmentation of all lacunae spaces belonging to a specific tessera and the separation of these spaces into individual lacunae required recently developed, novel tools.
• For error correction, we developed an interactive method that allowed us to quickly split lacunae that were accidentally merged, and to merge lacunae that were wrongly split.
• Finally, the tesserae and their corresponding lacunae were subdivided into structural wedges (i.e. specific anatomical regions) using a semi-manual approach.

With this processing pipeline, analysis of a variety of interconnected structural networks (e.g. vascular or lacuno-canalicular networks) can be achieved in a comparatively high-throughput fashion. In our study system, we were able to efficiently segment more than 12,000 lacunae in high-resolution scans of nine tesserae, providing a robust data set for statistical analysis.

## Graphical abstract

**Figure:**
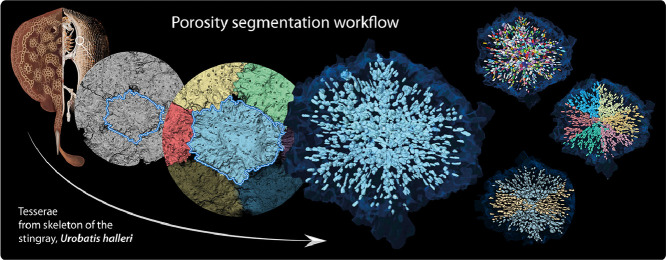

**Table:** Specifications Table

Subject Area:	Computer Science
More specific subject area:	Visual data analysis
Method name:	The current method is a combination of standard/published techniques and novel ones. The standard/published techniques (e.g. the watershed algorithm) are cited in the manuscript
Name and reference of original method:	The current method is a combination of standard/published techniques and novel ones. The standard/published techniques (e.g. the watershed algorithm) are cited in the manuscript
Resource availability:	Most tools are available in the commercial version of the Amira software.Additional custom Amira modules that were implemented for data processing can be obtained upon request from the corresponding author, as mentioned in text

## Introduction

**Fig. 1:**
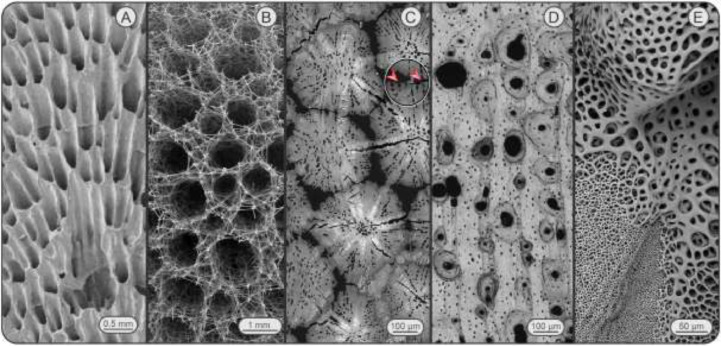
**Porosity in mineralized biological tissues. (**A) Sectioned (internal) view of a blue coral skeleton (*Heliopora coerulea*; Helioporacea). (B) Skeletal growth front of a reef-forming glass sponge (*Aphrocallistes vastus*; Hexactinellida). (C) A section through several tesserae from the skeleton of a stingray (*Urobatis halleri*; Chondrichthyes), the study model in the current and companion work [6]. The black gaps between tesserae are unmineralized joints, the small black dots within tesserae are cell lacunae. Note the large sample preparation cracks and the several instances of cell lacunae communicating to the exterior of tesserae (e.g. red arrows) – both situations would pose challenges to traditional segmentation protocols. (D) Osteonal bone from a dog femur (*Canis familiaris*; Carnivora). The larger cavities are vascular channels, the smaller ones peppering the matrix are cell lacunae. (E) The surface of an ossicle from a brittle star (*Ophiopteris papillosa*; Ophiuroidea). Note the large range of sizes and morphologies for porosities, both within and among images. All images are SEM ((C), and (D) from backscatter SEM). (A), (B), and (E) courtesy of James Weaver, (D) courtesy of Ron Shahar.

Porosity is a characteristic feature of mineralized biological tissues, from the skeletons of corals, sponges and radiolaria to the bone and dentin of vertebrates [1], [2], [3], [4], [5] (Fig. 1). These diverse tissues are perforated by canals and cavities of a huge range of size scales, from ostia, medullary cavities and foramina visible to the naked eye down to micron-scale tubules and passages and interstitial nanoscale porosities within the collagen-apatite matrix of teeth and bones. Passages or chambers can communicate to the exterior of the tissue or be bounded and entirely internal, can exhibit relatively uniform geometric properties or a range of constrictions and expansions, can be aligned in simple arrays or in complex and interconnected networks. Internal porosity can play mechanical roles (e.g. reducing weight, aiding buoyancy), but also physiological ones, providing pathways for nerves, vasculature and cell connections. The latter explains why 3D porosity organization and pore size-scale distribution is a vital consideration in tissue engineering scaffolding; see e.g. [4].

The characterization of biological porosities can be greatly challenged by their morphology (e.g. the degree of interconnectedness and linking to the exterior). Here, we describe the design and implementation of a processing pipeline allowing extraction and downstream quantification from microCT data of the lacuno-canalicular network (LCN) of tesserae, porous mineralized tiles that cover the cartilage skeletons of sharks and rays [6], [7], [8], [9] (Fig. 1(C)). The overarching goal of the pipeline is to efficiently segment multiple tesserae and their corresponding cell lacunae (several hundred per tessera) to carry out statistically-relevant quantitative analyses on a large scale. The tesseral LCN presents several generally-applicable segmentation challenges: (1) the tesserae are in close contact; (2) the LCN communicates to the exterior of the tesserae (i.e. complicating determination of the ends of passages); (3) the LCN exhibits serial constrictions (canaliculi) and expansions (cell lacunae) that we wished to analyze separately from one another.

The pipeline was developed for a companion study [6], which aimed to characterize the shapes, orientations, and spatial organization of the cell lacunae in tesserae (gaps where cells reside). To achieve this goal, individual cell lacunae had to be separated from one another with high fidelity, requiring us to solve several smaller segmentation problems. First, we had to segment out individual tesserae from the image data. Second, for each tessera, the entire LCN (i.e. all cell lacunae and canaliculi) had to be extracted, from which subsequently the individual cell lacunae needed to be separated. Finally, in order to allow study of the spatial arrangement and orientation of the cell lacunae with regard to their position in the skeleton (e.g. in association with neighboring tesserae), the cell lacunae needed to be divided into regions called ‘wedges’ [6]. Apart from the last step, these segmentation tasks can be grouped into three broad categories: (1) intensity value-based segmentation; (2) distance-based object separation; (3) cavity segmentation. Some tools that fall into these three categories are reviewed in the following paragraphs.

An important standard tool for intensity value-based segmentation is the watershed algorithm [10]. The basic watershed algorithm starts from local minima and floods the whole image, separating it into as many regions as there are local minima. Segmentation using this method usually results in what is called over-segmentation, because it separates the image in too many regions or segments (i.e. more than the actual number of objects of interest). A typical reason for over-segmentation is noise in the scan data, which can result in many local minima. Such local minima, however, can also be due to small substructural components of the material to be segmented. The hierarchical watershed algorithm [11] was developed to overcome such over-segmentations. It allows merging of neighboring regions according to several criteria. Hierarchical watershed is also similar to the contour-tree segmentation [12] with the major difference being that the latter starts from local maxima instead of local minima as the watershed does. If the number of objects to be segmented in an image is rather small, the marker-based watershed algorithm represents an efficient alternative since it allows the user to specify regions by manually setting a few seeds (starting points). In our processing pipeline, we apply marker-based watershed to segment out the individual tesserae (Section "*Segmentation of cell lacunae*" below).

When objects cannot be separated from one another by considering image intensities alone and are connected by extensions that are substantially narrower than the objects to be separated, distance-based object separation can be applied. The first step is usually to create a binary segmentation containing all objects of interest in the foreground. Then, a *distance transform* [13] is computed on the foreground resulting in an intensity image that can be segmented using the watershed or contour-tree segmentation algorithm. The standard distance transform is the *Euclidean distance transform* [14] that computes for each foreground voxel the shortest distance to any background voxel. One deficiency of this distance transform is its susceptibility to background noise. An alternative distance transform is the more recently developed *random-walk distance transform* [15] that computes for each foreground voxel the average length of all random walks starting at this foreground voxel and ending in any background voxel. This distance transform is much less prone to background noise and, hence, often results in superior segmentations when used in combination with watershed or contour-tree segmentation. In our processing pipeline, we apply the random-walk distance transform together with contour-tree segmentation to separate the individual cell lacunae from one another (Section "*Segmentation of cell lacunae*" below).

Another problem that often arises in image analysis of porosities is the determination of the border of a cavity space of interest to allow its separation from the 'real' background space (i.e. regions external to the scanned object). This problem occurs if the cavity space of an object is connected to the outside of the object, as in the tesserae LCN, where cell network passages connect to the exterior of tesserae [6,9] (Fig. 1(C)). To solve this problem, the *ambient occlusion algorithm* [16] was developed that computes an intensity field that assigns the degree of occlusion from ‘simulated' ambient light to each voxel. In our processing pipeline, we apply this algorithm to separate the tessera cell lacunae space from the background space outside of the tesserae.

## Specimen preparation and SR–**µ**CT scanning

Detailed descriptions of sample preparation and scanning protocols are provided in [6]; we provide abridged versions here. Samples of the propterygium (a long, rod-like portion of the skeleton, supporting the wing) were dissected from two adult Haller's round rays (*Urobatis halleri*)—a 19 cm disk width (DW) female and a 21.4 cm DW male. *Urobatis halleri* is an established study system for tessellated cartilage biology, with the majority of recent high-resolution, ultrastructural data coming from this species (e.g. [7,^9^,[17], [18], [19]). Long strips of tessellated cartilage were excised from skeletal samples, air-dried and affixed upright in micro-centrifuge tubes for subsequent synchrotron experiments.

**Fig. 2:**
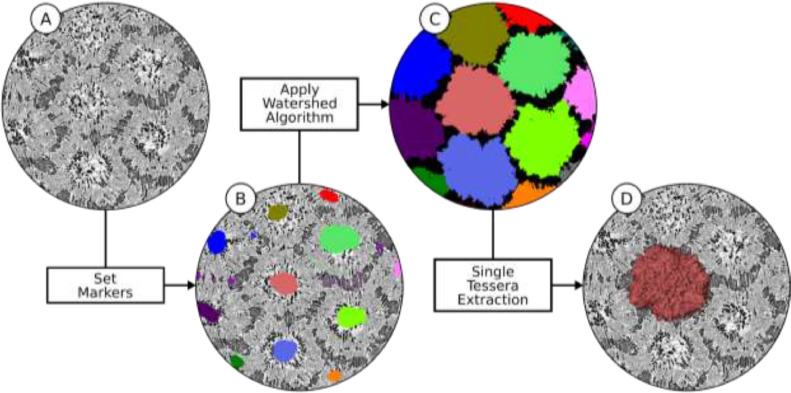
**Pipeline for tesserae segmentation.** (A) Input µCT slice, (B) seed markers, (C) separated tesserae after applying the watershed algorithm, (D) volume rendering of a single tessera. Tesserae shown in (A), (B) and (D) are mineralized and therefore exhibit higher (lighter) intensity values; the joints between tesserae and the cell lacunae inside tesserae are darker.

Tesserae samples were scanned in synchrotron radiation micro-computed tomography (SR-μCT) at the BAMline, BESSY II synchrotron source, Helmholtz-Zentrum Berlin für Materialien und Energien (HZB) and reconstructed, as described in [6]. The resulting data sets contained several tesserae in close contact, with effective pixel sizes of 876 nm (Fig. 2).

## Processing pipeline

Here, we describe the processing steps performed to segment tesserae and their cell lacunae, as well as the separation of the tesserae and their cell lacunae into structural wedges (i.e. specific anatomical regions). These steps are the prerequisite to the lacuna morphometric analysis described in the Methods in [6]. All image and geometry processing described in this section was carried out in the visualization software Amira (AmiraZIBEdition 2019.12) [20]. The Amira modules used for data processing and analysis are detailed below. Whereas most are available in the commercial version of the software, for some steps, we implemented custom Amira modules. These can be obtained upon request from the corresponding author.

The processing pipeline consists of three major steps: (1) the segmentation of all individual tesserae in each data set; (2) the segmentation of individual cell lacunae; and (3) the grouping of cell lacunae according to the tessera wedges. These three steps are described in detail below.

### Segmentation of tesserae

For the segmentation of the input data set (Fig. 2(A)) into individual tesserae (Fig. 2(C)), a marker-based watershed transformation was used [10]. This technique involves the manual placement of initial markers in distinct regions (e.g. individual tesserae, joint spaces; Fig. 2(B)) to act as seeds from which segmentation will begin. This was performed using Amira's segmentation editor, the software's primary segmentation tool. The watershed algorithm expands outward from the markers until the entire data set is segmented, with any remaining regions between tesserae belonging to the background (label/material value = 0) (Fig. 2(C)). In addition to the markers, we used an ‘edge image’ generated from the original intensity field. This is a very common approach and serves to guide the algorithm's detection of material boundaries. In the current study, the edge image was generated by using the Watershed tool of Amira's segmentation editor. Edges appear in places with a rapid change of intensity values. As a result, the strength of an edge indicates the likelihood of a material boundary.

**Fig. 3:**
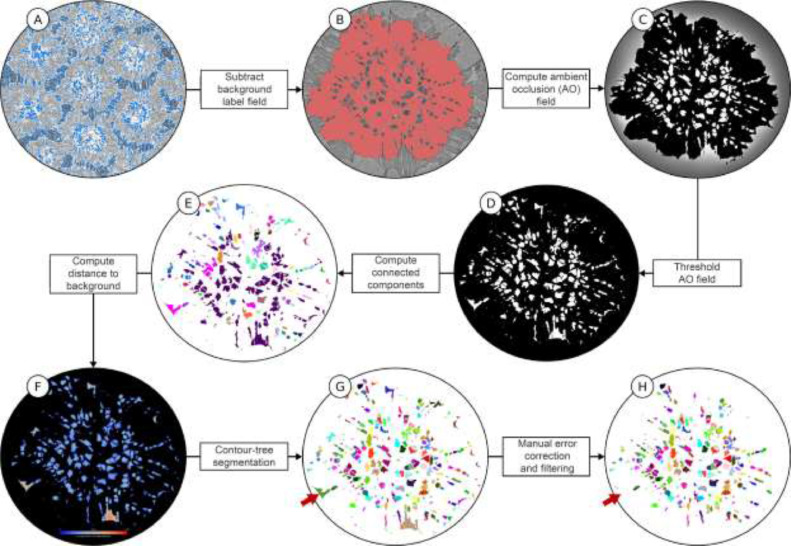
**Segmentation pipeline for cell lacunae.** (A) Intratesseral (cell lacunae) and intertesseral space (regions outlined in blue), computed using local thresholding (note, the region is zoomed out relative to images (B)-(H) to show multiple tesserae), (B) single tessera, excluding its cell lacunae, generated by subtracting the label field of (A) from the tessera label generated in the previous tessera segmentation step (see Fig. 2), (C) ambient occlusion (AO) field, (D) binary label field of AO, generated from AO field in (C), (E) separation of disconnected cell lacunae, (F) average length field, (G) contour-tree segmentation, (H) removal of objects incorrectly interpreted as cell lacunae.

From the watershed segmentation result (Fig. 2(D)), tesserae were extractable as separate data sets, facilitating the downstream segmentation of cell lacunae within individual tesserae. Each label of the watershed segmentation result, however, represented a segmented tessera including all of its internal spaces (i.e. cell lacunae were not yet isolated from the tessera label field) (Fig. 2(C) and (D)). Furthermore, it is important to note that the lacuno-canalicular passages within tesserae often communicate to the outside (i.e. into the intertesseral joint space; [7]) (some of such cell lacunae are visible in Fig. 3(B) and (C)). As a result, the borders of this watershed segmentation result did not yet enclose all cell lacunae ‘belonging’ to a given tessera, but rather partially lacked those lacuno-canalicular passages open to the background, thus requiring additional steps described below.

### Segmentation of cell lacunae

The segmentation of the tesseral LCN and its subsequent division into individual cell lacunae required several steps that are explained in detail below.

#### Separation of cell lacunae from background

To extract the cell lacunae within a tessera, all voxels representing background unmineralized tissue (i.e. voxels with lower gray values) were first segmented in the original data set using a local threshold, and then stored as a separate label field (Fig. 3(A)). Subsequently, this background label field was subtracted from each tessera's label field, resulting in a label field with the tessera (mineralized material) as foreground and the tessera's internal spaces (including its cell lacunae) and the area external to the tessera as background (Fig. 3(B)).

#### Ambient occlusion field

As stated above, it is challenging to define all cell lacunae (i.e. background voxels) that ‘belong’ to a tessera, due in particular to those regions where the tessera lacuno-canalicular network is open to the outside [7]. This problem is akin to that of defining the inside/outside borders of structures with irregular openings (e.g. caves). To avoid ‘losing’ cell lacunae to the surrounding background, an ambient occlusion scalar field was calculated from the previous result. In this algorithm [16], rays are cast from each background voxel through the label field in all directions. The ratio of the number of rays striking the foreground (i.e. the tessera) to the total number of rays defines the ambient occlusion value (Fig. 3(C)). In this way, the algorithm allows the identification of background voxels surrounded by foreground voxels (e.g. cell lacunae surrounded by mineralized tissue, but open at one end to the background). By applying a threshold to the resultant ambient occlusion field that accounts for all cell lacunae (i.e. background voxels) belonging to the tessera, a binary cell lacunae label field is generated (Fig. 3(D)).

#### Connected components

To divide the single label produced in the previous step containing all lacunae into multiple, individual lacuna labels, the connected components algorithm was applied. This algorithm searches for regions of contiguous voxels in the binary label field, defining each as an individual object (i.e. assigning each to a new label ID). At this point, any isolated cell lacunae (i.e. those not linked to other cell lacunae) were identified as individual objects. However, any multi-lacunae objects (i.e. cell lacunae connected by canaliculi) still required disarticulation (Fig. 3(E)).

#### Contour-tree segmentation

The contour-tree segmentation [12] was used for the remaining separation of connected cell lacunae. This algorithm used the random-walk distance transform [15] that, for each voxel of the result of the previous step, calculated the average length of all random walks from this voxel to the background (Fig. 3(F)). The *Random-Walk Distance Transform* is implemented as a custom Amira module. It takes as input the binary label field of all cell lacunae and outputs a scalar field containing the random-walk distance to the background from each voxel of the cell lacunae. The module does not require any parameter. The *Contour-Tree Segmentation* module takes as input the random-walk distance field and a single parameter, the persistence value, that defines the degree of merging. The contour-tree segmentation using the random-walk distance field takes advantage of the ‘string-of-pearls’ appearance of objects comprised of multiple cell lacunae connected by canaliculi (i.e. spheroidal objects connected by narrow, short links; [6,9]), splitting the multi-lacunae labels at their narrowest points (i.e. their canaliculi). The result is shown in Fig. 3(G).

#### Manual error correction and filtering

As a final step, the label field was cleaned and refined by removing objects that had been wrongly interpreted as cell lacunae in the segmentation (Fig. 3(H)). First, objects with volumes <70 µm^3^ (far smaller than that of cell lacunae; [6]) were considered as noise and deleted. This was performed in Amira using the *Label Analysis* module followed by application of the *Filter Analysis* module. Additionally, objects considerably larger than cell lacunae were manually removed after being verified as errors by comparison with the raw grayscale data. These were also readily distinguishable from actual cell lacunae by their morphologies, typically being either crack artifacts in the sample or invaginations in the joint face at the tesseral edge (e.g. as indicated by the red arrow in Fig. 3(G) and (H)). We applied a custom Amira module that allowed us to select and remove such objects by directly picking the labels either on an *Orthoslice* or the *Voxelized Volume Rendering* visualization. Alternatively, a somewhat slower combination of *Arithmetic* module and *Quick Probe* tool could be utilized. Following this, any remaining passages (canaliculi) connecting cell lacunae were removed (i.e. isolating individual cell lacunae). This was achieved using another custom Amira module that allowed one to specify a single label and the number of cell lacunae into which the label should be split. The module again exploited distance-based object separation using the random-walk distance transform and the contour-tree segmentation. First, the random-walk distance transform was computed on the single specified label. Subsequently, the contour-tree segmentation was run and an adequate persistence value was automatically identified that separated the label into the desired number of cell lacunae.

### Subdivision of tesserae cell lacunae into wedge data sets

**Fig. 4:**
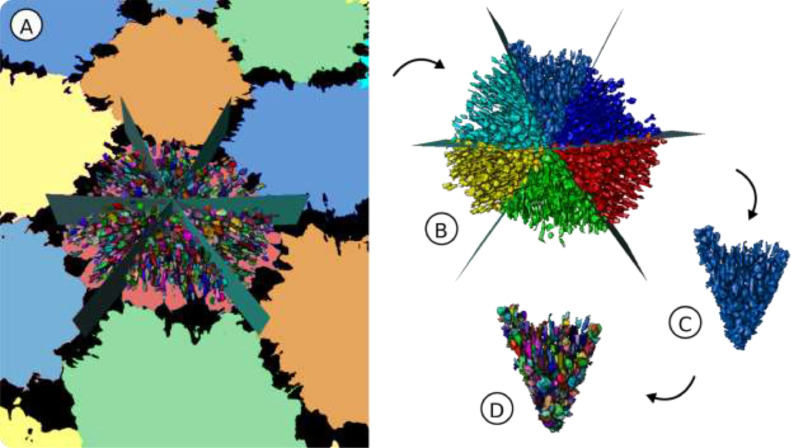
**Subdivision of the lacuna label field.** (A) A single tessera and its neighbors, with the focal tessera's segmented cell lacunae subdivided into wedges by planes, (B) division of the entire lacunar data set and assignment into wedges, (C) extraction of one wedge, (D) generation of new (i.e. wedge-specific) lacuna IDs.

Previous work on tesserae ultrastructure has demonstrated the presence of large, linear arrays of collagen fibers linking adjacent tesserae, with cell lacunae following the predominant fiber orientation (e.g. [7,^9^,^17^,19], Fig. 2(A)). In polarized light microscopy, these fiber arrays appear to converge on the center of tesserae [7,[21], [22], [23]]. As a result, we hypothesized that cell lacunae orientation is influenced by neighboring tesserae, particularly the further cell lacunae are from the center of their host tessera [6]. In order to investigate this theory—specifically, whether cell lacunae are oriented in a direction perpendicular to the joint face with the nearest neighboring tessera—the cell lacunae label field resulting from the segmentation workflow (e.g. Fig. 3(H)) was further subdivided into ‘wedges’ (Fig. 4). These wedges are triangular regions, with their vertices at the tessera center and their bases at the tessera edge (see [6]).

To subdivide the segmented cell lacunae into individual wedges, a semiautomatic custom Amira module, *Tesserae Wedges*, was developed. As input, this module requires the binary label field of a single tessera and its corresponding cell lacunae label field. The center of that label field and its associated local coordinate system can either be computed directly from the module or can be given as optional input. The center of the tessera was calculated from the tessera label field by averaging the position of all voxels belonging to the tessera. The local tessera coordinate system was calculated from the tessera label field via principal component analysis (PCA). From the center point and the first and second principal axes of the PCA, the module created sectioning planes (Fig. 4). The number of planes was set in order to divide each tessera into as many wedges as the tessera had neighbors (e.g. the tessera in Fig. 4 has six neighbors and is divided by six planes); the anatomical justification for this choice is explained in [6]. Wedge sectioning planes could be manually rotated; the sectioning planes were positioned to pass through the tesserae triple junctions—the intersection points of three neighboring tesserae (Fig. 4(A))—thereby defining the zones of interaction between a tessera of interest and its neighbors. Once the sectioning planes were set, the cell lacunae label field was divided into wedges accordingly (Fig. 4(B)). For those cell lacunae bisected by a sectioning plane, their wedge assignment was decided by the position of the lacuna center of mass. Lastly, in order to facilitate the wedge-wise analysis of cell lacunae, all cell lacunae in each wedge were extracted into separate data sets (Fig. 4(D)). Following this step, the coordinate axes for each wedge were calculated and cell lacunae morphology and orientation quantified, as described in the Methods in [6]. Calculation of the cell lacunae morphometric variables was accomplished with a custom analysis module, combining both common Amira analysis variables and additional variables specific to our research questions; see Methods and Table 1 in [6] for more details.

## Conclusions

We have presented an effective segmentation pipeline that makes use of standard segmentation methods like the watershed algorithm, but also uses more advanced, newly developed tools like the ambient occlusion algorithm and the random-walk distance transform. Marker-based watershed segmentation, which we used for the segmentation of the tesserae, is a very powerful tool when the segmentation of a small to medium number of objects is required. For very large numbers of objects (e.g. in the hundreds to thousands), however, the hierarchical watershed algorithm or its kin, the contour-tree segmentation, should be used. We exploited the contour-tree segmentation for the separation of the cell lacunae, which we applied to the result of the random-walk distance transform of the binary cell lacunae segmentation. This new distance transform resulted in a much better initial segmentation compared to using the more traditional Euclidean distance transform, leading to fewer segmentation errors and, thus, drastically reducing the manual work required for error correction. Instead of developing a fully automated segmentation workflow, we favored some degree of manual user control over a completely automated solution that would have required substantially more time for implementation. For example, we used an interactive approach to correct falsely split cell lacunae, rather than spending significant time refining the automated segmentation. Furthermore, instead of implementing a fully automated approach for the subdivision of tesserae into wedges, we used a semiautomatic approach in which the planes separating wedges were manually determined by the user. We believe that such combinations of automated and interactive segmentation methods produce efficient and reliable results for many analysis problems. These considerations are relevant to the segmentation of many complex biological structures, and so are particularly important for investigations of biological porosity and network structure, which rely increasingly on high-throughput analyses of large data sets (e.g. synchrotron microCT and FIB-SEM tomography volumes; see e.g. [5,15]).

## Declaration of Competing Interest

The authors declare that they have no known competing financial interests or personal relationships that could have appeared to influence the work reported in this paper.
